# Hypoparathyroidism following total thyroidectomy: high rates at a low-volume, non-parathyroid institution

**DOI:** 10.3389/fendo.2024.1330524

**Published:** 2024-01-18

**Authors:** Ali Abood, Therese Ovesen, Lars Rolighed, Frédéric Triponez, Peter Vestergaard

**Affiliations:** ^1^ Department of Otorhinolaryngology, Goedstrup Hospital, Herning, Denmark; ^2^ Department of Otorhinolaryngology, Head- and Neck Surgery, Aarhus University Hospital, Aarhus, Denmark; ^3^ Thoracic and Endocrine Surgery Division, Geneva University Hospital, Geneva, Switzerland; ^4^ Department of Endocrinology, Aalborg University Hospital, Aalborg, Denmark; ^5^ Steno Diabetes Center North Denmark, Aalborg University Hospital, Aalborg, Denmark

**Keywords:** thyroidectomy, hypoparathyroidism, hypocalcemia, parathyroid damage, low-volume, surgeon experience

## Abstract

**Background:**

Hypoparathyroidism following total thyroidectomy is globally the most common complication to thyroid surgery. The reported complication rates vary widely and might be highly dependent on the surgical experience. In this study we aimed to evaluate the rate of hypoparathyroidism following primary total thyroidectomy at a low-volume institution that only performs thyroid surgery and does not have any experience with parathyroid surgery.

**Methods:**

Retrospective cohort study. All patients undergoing primary total thyroidectomy at the ENT-Department, Goedstrup Hospital, Denmark, over a 5-year period (2016-2020) were identified through the procedure codes for total thyroidectomy. Medical records, pathology reports, biochemical and medical histories were fully assessed for each patient. The primary endpoint was the rate of hypoparathyroidism- both immediate and permanent. Secondary outcomes were parathyroid gland identification rates, rates of parathyroid gland autotransplantation, and rates of inadvertent parathyroid gland excision.

**Results:**

A total of 89 patients were included in the final analysis. A total of 33 patients (37.1%) experienced immediate hypoparathyroidism following surgery, while 30 patients (33.7%) still were on active vitamin D two months postoperatively. One year following surgery, 28 patients (31.5%) were still on active vitamin D and were considered as having permanent hypoparathyroidism. Sixty-one percent of the parathyroid glands were identified intraoperatively, and 19% of the patients experienced parathyroid autotransplantation. Inadvertent parathyroid gland excision occurred for 21% of the patients and was associated with a significantly increased risk of permanent hypoparathyroidism (RR = 2.99; 95% CI: 1.36 – 6.62, p = 0.005).

**Conclusion:**

Both transient and permanent hypoparathyroidism following total thyroidectomy at a low-volume, non-parathyroid institution occurred with much higher frequencies than previously reported. The elevated rates were most likely due to the low-volume, non-parathyroid nature of the surgeons which in part was mirrored in low parathyroid gland identifications rates, and high rates of autotransplantation and inadvertent parathyroid gland excision.

## 1 Introduction

Hypoparathyroidism (hypoPT) is a common complication to total thyroidectomy on a global scale (1, 2). The condition is a consequence of intraoperative damage to the parathyroid glands (PGs) or their vascular supply, resulting in an impaired parathyroid hormone (PTH) secretion and subsequent hypocalcemia (3). On the short term, this can lead to paraesthesias, muscle spasms and seizures (4). If the condition persists and the PG function is not restored, the condition becomes permanent. It is well-described that permanent hypoPT is associated with reduced quality of life (5–9). In addition, novel studies have shown that permanent hypoPT following total thyroidectomy also might be associated with an increased risk of cardiovascular events, renal insufficiency, malignancy as well as an increased mortality (10, 11). Thus, the severity of permanent hypoPT is potentially of a much larger magnitude than previously assumed.

Although common, hypoPT following total thyroidectomy rarely becomes permanent (1, 2). However, recently several studies have reported much higher rates of permanent hypoPT, reaching up to 36% (12–18). This can in part be ascribed to differences in the definition of permanent hypoPT (19, 20). Another potential explanation for this variety, can be the fact that the surgical procedures are performed by different types of surgeons (16, 18, 21, 22). Kiernan et al. (22) have shown that more experienced surgeons are better at finding PGs intraoperatively, while Chadwick (21) and Anneb*ä*ck et al. (18) report higher rates of postoperative hypocalcemia when total thyroidectomy is performed by low-volume surgeons. In the same line, Reinke et al. (16) have shown lower rates of postoperative hypoPT when total thyroidectomy is performed by surgeons who perform both thyroid and parathyroid surgery at a tertiary health care institution. Surgeons performing only thyroid surgery on the other hand, had significantly higher rates of postoperative hypoPT. All together, these studies suggest that actual rates of hypoPT at low-volume institutions that only perform thyroid surgery might be even higher, which indeed would be critical. To our knowledge, data from such institutions do not exist in the current literature.

In this study we aimed to evaluate the rates of hypoPT following total thyroidectomy and identify potential risk factors for hypoPT at a low-volume institution that only performed thyroid surgery and had no experience with parathyroid surgery.

## 2 Materials and methods

The study was designed as a retrospective cohort study at the ENT-Department, Goedstrup Hospital, Denmark. The entire Department performs approximately 130 thyroidectomies annually, most of which being hemithyroidectomies (80%). Surgery is only performed on benign indications. The surgeon with the highest surgical volume performs less than 10 total thyroidectomies and approximately 30 hemithyroidectomies per year. Parathyroid surgery is not performed at the Department. Thus, it is considered a low-volume (23–26), non-parathyroid institution.

All patients undergoing primary total thyroidectomy over a 5-year period (from 01.01.2016 to 31.12.2020) were identified through the procedure codes for total thyroidectomy (KBAA25 and KBAA60). Patients with a history of previous thyroid surgery were excluded. Medical records, pathology reports, biochemical and medical histories were fully assessed for each patient. The primary end-point was the rate of postoperative hypoPT- immediate as well as permanent. Secondary outcomes were PG identification rates, rates of PG autotransplantation, and rates of inadvertent PG excision.

### 2.1 Definitions

Immediate hypoPT was considered, when postoperative PTH levels were low and resulted in hypocalcemia that required treatment with active vitamin D. Likewise, permanent hypoPT was defined as a condition where the PTH secretion was insufficient to maintain normal plasma calcium levels without treatment with active vitamin D one year following surgery. In practice, this meant that a diagnosis of permanent hypoPT was given if there was a need for treatment with active vitamin D one year following surgery. In contrast, permanent hypoPT was ruled out if the patient had normal plasma calcium and PTH levels without the need for active vitamin D one year following surgery. If a patient with immediate hypoPT regained sufficient parathyroid function within the first postoperative year, the condition was defined as transient hypoPT. Normocalcemia was defined as plasma ionized calcium concentrations in the normal range (1.18 – 1.32 mmol/L). The normal range for plasma PTH was 1.6 – 6.9 pmol/L.

### 2.2 Surgical procedures

Each surgical procedure was performed by two surgeons – a first operator and an assistant. The assistant could either be a thyroid surgeon or a resident. No devices for PG visualization were used in any case. Nerve monitoring was used routinely and magnifying loupes were used upon surgeon’s choice. From the surgery and onwards, all patients were routinely given prophylactic calcium and vitamin D3 supplementations (800 mg of calcium and 38 µg vitamin D3) in accordance with local and regional guidelines. This was done regardless of the levels of PTH and calcium, as some studies have shown beneficial effects of prophylactic use (27–29). Patients were only discharged from the Department when ionized calcium (Ca^2+^) plasma levels were stable (with or without active vitamin D treatment).

### 2.3 Follow-up

On postoperative day 1 (POD1), recurrent laryngeal nerve (RLN) function was routinely assessed by flexible laryngoscopy, and PTH and ionized calcium were measured in each case. After discharge, patients had different follow-up programs according to their biochemical status. Patients with immediate hypoPT were followed more closely with frequent blood tests. Normoparathyroid patients were not followed as close with some patients having their first postoperative biochemical control several months postoperatively. As all patients had their plasma levels of PTH and ionized calcium controlled minimum two months following surgery, this was defined as the first biochemical follow-up in the study. Patients who still received active vitamin D treatment and/or were hypocalcemic at that point, were followed until one year postoperatively (POY1). If patients still required treatment with active vitamin D at that point, they were considered as having permanent hypoPT and were not further evaluated in this study. Their follow-up from that point was carried out by the Department of Endocrinology.

### 2.4 Statistics

Continuous data were evaluated by either mean values and 95% confidence intervals or medians and interquartile ranges, depending on the distribution. Comparisons were made using a t-test for normally distributed data, and Mann-Whitney’s test for non-normally distributed data. Binary data were compared through Fisher’s exact test or Chi2-test. A two-tailed p-value of <0.050 was considered statistically significant. STATA 18.0 SE was used for data analysis.

## 3 Results

**Figure 1:**
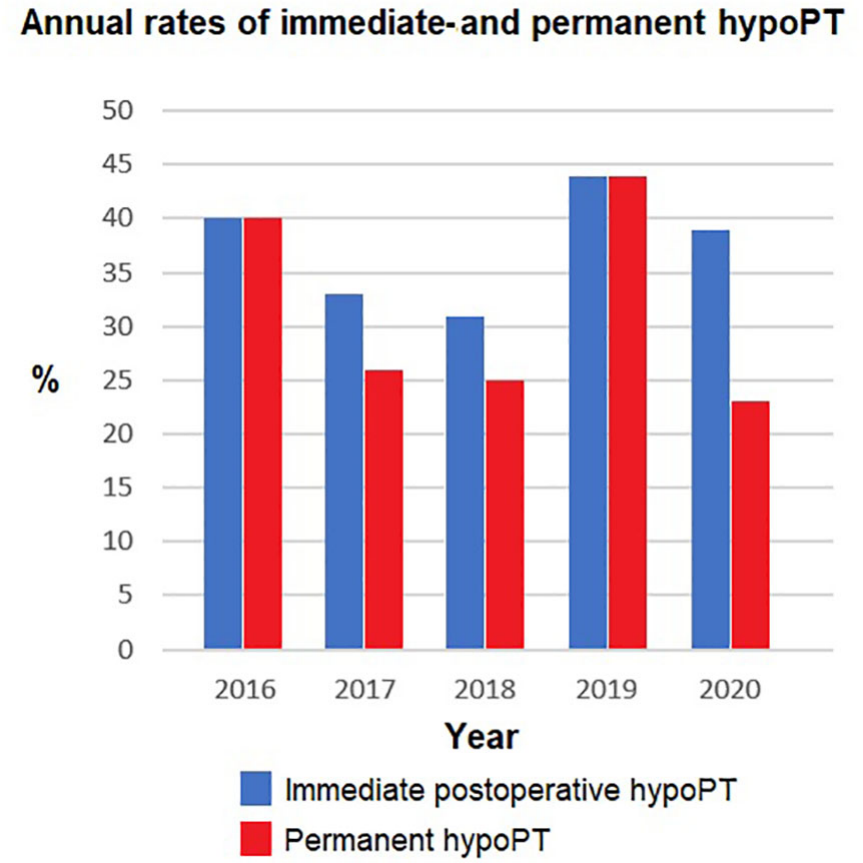
Annual rates of both immediate and permanent hypoPT following total thyroidectomy. The annual rates were compared using Fisher's exact test. No statistically significant difference was found, p < 0.050.

A total of 94 patients were initially identified through the procedure codes for total thyroidectomy. Five patients had previously undergone thyroid surgery and were excluded. Thus, the final cohort consisted of 89 patients who underwent primary total thyroidectomy. Six different surgeons performed the surgeries over the 5-year period. The mean age at the time of surgery was 49.7 years (95% CI: 47.0 – 52.4) and 87.6% were female (**Table 1**). The majority of patients underwent surgery due to either benign goiter with compressive symptoms (42.7%) or Graves’ disease (41.6%). A total of 33 patients (cumulated incidence proportion: 37.1% - 95% CI: 27.8 - 47.5%) experienced immediate hypoPT following surgery, while 30 patients (33.7% - 95% CI: 24.7 - 44.0%) still were on active vitamin D two months postoperatively. One year following surgery, 28 patients (31.5% - 95% CI: 22.8 – 41.7%) were still on active vitamin D and were considered as having permanent hypoPT (**Tables 1**, **2**; **Figure 1**). Permanent hypoPT rates per surgeon did not differ significantly (**Table 1**).

**Table 1:** Characteristics of included patients.

	Total	Permanent hypoPT		*p-value*
		No	Yes	
No. of patients, *n*	89	61 (68.5)	28 (31.5)	
Age, *mean (95% CI)*	49.7 (47.0 – 52.4)	49.7 (46.7 – 52.8)	49.7 (44.0 – 55.4)	1.000 ^a^
Sex, female, *n (%)*	78 (87.6)	56 (91.8)	22 (78.6)	0.093 ^b^
Body Mass Index (BMI), *mean (95% CI)*	29.1 (27.7 – 30.4)	29.9 (28.1 – 31.6)	27.4 (25.2 – 29.7)	0.099 ^a^
Indication for surgery				
Goiter, *n (%)*	38 (42.7)	28 (45.9)	10 (35.7)	0.489 ^b^
Graves’ disease, *n (%)*	37 (41.6)	22 (36.1)	15 (53.6)	0.165 ^b^
Thyrotoxicosis, *n (%)*	14 (15.7)	11 (18.0)	3 (10.7)	0.535 ^b^
Surgery-related outcomes				
No. of PGs identified, *n (%)*	218 (61.2)	152 (62.3)	66 (58.9)	0.560 ^b^
PG autotransplantation, *no.of patients (%)*	17 (19.1)	10 (16.4)	7 (25)	0.390 ^b^
Weight of specimen, *median (IQR)*	96 (45-181)	89 (36 – 181)	107 (69 – 186)	0.149 ^c^
Length of stay due to hypocalcemia in direct relation to surgery, days, *mean (95% CI)*	3.1 (2.8-3.4)	2.5 (2.3 – 2.8)	4.3 (3.7 – 4.8)	<0.010 a^,*^
No. of surgeries performed by Surgeon 1, *n (%)*	40 (44.9)	26 (65.0)	14 (35.0)	> 0.050 ^bb^
No. of surgeries performed by Surgeon 2, *n (%)*	20 (23.6)	16 (80.0)	4 (20.0)	
No. of surgeries performed by Surgeon 3, *n (%)*	18 (20.2)	12 (66.7)	6 (33.3)	
No. of surgeries performed by Surgeon 4, *n (%)*	8 (9.0)	6 (75.0)	2 (25.0)	
No. of surgeries performed by Surgeon 6, *n (%)*	2 (2.2)	1 (50.0)	1 (50.0)	
No. of surgeries performed by Surgeon 5, *n (%)*	1 (1.1)	0 (0.0)	1 (100.0)	
Complications				
Immediate RLN injury, *n (%)*	11 (6,2)	6 (4.9)	5 (8.9)	0.325 ^b^
Permanent RLN injury, *n (%)*	5 (2.8)	3 (2.5)	2 (3.6)	0.650 ^b^
Postoperative bleeding, *n (%)*	4 (4.5)	2 (3.3)	2 (7.1)	0.587 ^b^
Postoperative infection, *n (%)*	3 (3,4)	2 (3.3)	1 (3.6)	1.000 ^b^
Biochemistry				
P-PTH POD1 < 1.6 pmol/L, *n (%)*	45 (49.4)	17 (27.9)	28 (100)	<0.010 ^b,*^
P-Ca^2+^ POD1 < 1.18 mmol/L, *n (%)*	57 (64.0)	29 (47.5)	28 (100)	<0.010 ^b,*^
P-PTH at first follow-up < 1.6 pmol/L, *n (%)*	14 (15.7)	0 (0.0)	14 (50.0)	<0.010 ^b,*^
P-Ca^2+^ at first follow-up < 1.18 mmol/L, *n (%)*	19 (21.3)	1 (1.6)	18 (64.3)	<0.010 ^b,*^
P-PTH POY1 < 1.6 pmol/L, *n (%)*	15 (16.9)	0 (0.0)	15 (53.6)	<0.010 ^b,*^
P-Ca^2+^ POY1 < 1.18 mmol/L, *n (%)*	21 (23.6)	0 (0.0)	21 (75.0)	<0.010 ^b,*^
PG autotransplantation and P-PTH POD1 < 1.6 pmol/L, *n (%)*	8 (8.9)	1 (1.6)	7 (25.0)	<0.010 ^b,*^
Histology				
Colloid goiter, *n (%)*	51 (57.3)	33 (54.1)	17 (60.7)	0.648 ^b^
Hyperplasia, *n (%)*	29 (32.6)	21 (34.4)	8 (28.6)	0.634 ^b^
Inflammation, *n (%)*	4 (4.5)	2 (3.3)	2 (7.1)	0.587 ^b^
Riedels Thyroiditis, *n (%)*	1 (1.1)	1 (1.6)	0 (0.0)	1.000 ^b^
Follicular adenoma, *n (%)*	1 (1.1)	1 (1.6)	0 (0.0)	1.000 ^b^
Follicular carcinoma, *n (%)*	1 (1.1)	1 (1.6)	0 (0.0)	1.000 ^b^
Papillary carcinoma, *n (%)*	2 (2.2)	2 (7.1)	0 (0.0)	1.000 ^b^
Lymphoma, *n (%)*	1 (1.1)	0 (0.0)	1 (3.6)	1.000 ^b^
Inadvertently excised PGs, *n (%)*	23 (6.5)	9 (3.7)	14 (12.5)	0.004 ^b *^
Inadvertent PG excision, *no. of patients (%)*	19 (21.3)	8 (13.1)	11 (39.3)	0.011 ^b *^

^a^: Student’s t-test. ^b^: Fisher’s exact test. ^c^: Mann Whitney’s test. ^bb^: Comparison of permanent hypoPT rates among the six different surgeons by Fisher’s exact test. ^*^: Statistically significant.

**Table 2:** Detailed annual rates of immediate- and permanent hypoPT following total thyroidectomy.

	2016	2017	2018	2019	2020	Total
**No. of patients, *n***	*15*	27	16	18	13	89
**Immediate hypoPT**	6(40%)	9 (33.3%)	5 (31.3%)	8 (44.4%)	5(38.5%)	33 (37.1%)
**Permanent hypoPT**	6(40%)	7 (25.9%)	4 (25%)	8 (44.4%)	3(23.1%)	28(31.5%)

**Figure 1** shows the annual rates of both immediate- and permanent hypoPT over the 5-year period. The highest rate (44.0%) for permanent hypoPT was in 2019. The lowest rate (23.1%) occurred in 2020. There was no statistically significant difference in the annual rates of both immediate and permanent hypoPT when making a comparison between the different years, *p >*0.050.

Patients with permanent hypoPT were compared with patients with normal parathyroid function one year following surgery in terms of different characteristics (**Table 1**). Patients with permanent hypoPT more often experienced inadvertent PG excision than normoparathyroid patients (39.3% vs 13.1%, *p* = 0.010). In the same line, patients with permanent hypoPT also experienced more PG autotransplantations, although not statistically significant (25.0% vs 16.4%, *p* = 0.390). Overall PG identification rate was 61.2%. Although nonsignificant, patients with permanent hypoPT had slightly lower identification rates than normoparathyroid patients (58.9% vs. 62.3%, *p* = 0.560). Patients with Graves’ disease were overrepresented among patients with permanent hypoPT but again no statistical significance was obtained (53.6% vs. 36.1%, *p* = 0.165). Not surprisingly, patients with permanent hypoPT had a longer postoperative hospital stay than normoparathyroid patients (3.1 days (95% CI 2.8 -3.4) vs. 2.5 days (95% CI 2.3 – 2.8), *p* < 0.010). The rate of immediate recurrent laryngeal nerve injury and median weight of the excised specimen were both higher among patients with permanent hypoPT, although statistically insignificant (*p*=0.325 and *p*=0.149 respectively). No statistically significant differences in histology- or other complication-related outcomes were found when comparing the two groups.

**Table 3** shows that inadvertent PG excision is associated with a statistically significant relative risk (RR) of developing permanent hypoPT of 2.99 (95% CI 1.36 – 6.62, *p* = 0.005). Patients experiencing PG autotransplantation had a RR of permanent hypoPT of 1.41 (0.72 – 2.77, p = 0.340). Finally, patients with Graves’ disease had a RR of permanent hypoPT of 1.49 (0.92 -2.40, *p* = 0.120).

**Table 3:** Potential risk factors for permanent hypoPT.

	RR of permanent hypoPT	*p-value*
**Inadvertent PG excision**	2.99 (95% CI: 1.36 – 6.62)	0.005^*^
**PG Autotransplantation**	1.41 (95% CI: 0.72 – 2.77)	0.340
**Graves’ disease**	1.49 (95% CI: 0.92 – 2.4)	0.120

^*^: Statistically significant.

Analyzed using Chi2-test.

## 4 Discussion

HypoPT following total thyroidectomy occurred with remarkable high frequencies when surgery was performed at a low-volume institution that only performed thyroid surgery and did not have experience with parathyroid surgery. Over a 5-year period, the overall rate of immediate postoperative hypoPT was 37.1%, and 31.5% for permanent hypoPT. Especially the rate of permanent hypoPT was noticeably high in our study in comparison to others. Systematic reviews by Edafe et al. (1) and McMurran et al. (2) both reported permanent hypoPT rates between 0-3%, which is much lower than what was found in our study. In part, this can be explained by differences in the definition of permanent hypoPT. The definition used in our study is supported by recent international guidelines (30). However, we did not take regular calcium and/or vitamin D3 supplementation into consideration, as all patients routinely receive calcium/vitamin D3 supplementations from the time of surgery and onwards. Furthermore, the long-term morbidity and mortality shown to be associated with permanent hypoPT in recent studies, are based on a definition that is exclusively dependent on active vitamin D (10, 11). We therefore believe, that the definition used in our study is appropriate for the purpose.

Benmiloud et al. (31) reported a rate of permanent hypocalcemia of 1.3%. In that study, permanent hypocalcemia was defined as persisting hypocalcemia 6 months following surgery. However, approximately 66% of the patients received calcium and vitamin D postoperatively, and it is not known if their normocalcemia was a result of the medical treatment. If so, the rate of permanent hypocalcemia would have been higher according to the definition used in our study. On the other hand, Dip et al. (32) found that only 1.2% of patients required long term medical treatment with calcium and/or vitamin D following total thyroidectomy. In the same line, DiMarco et al. (33) reported that 0% of the patients were keen on medical supplementation to maintain normocalcemia postoperatively. These rates are more comparable to ours in terms of the definition of permanent hypoPT. Thus, despite the similarities in definitions, permanent hypoPT still occurred at much higher frequencies in our study. The fact that the mentioned studies were performed at high-volume institutions and/or institutions that also performed parathyroid surgery suggests that this complication is highly dependent on the surgical expertise. In other words, these findings suggest that permanent hypoPT rates might be particularly high at low-volume institutions that only perform thyroid surgery.

The same conclusion was made by Reinke et al. (16) in a recently published study. They found very high rates of permanent hypoPT at a tertiary health care institution that initially did not perform parathyroid surgery. After the introduction of parathyroid surgery to the department, and restriction of cases with Graves’ disease to only surgeons that also did parathyroid surgery, the rates of permanent hypoPT declined remarkably. From an objective point of view, it seems reasonable to assume that a thyroid surgeon that also has an extensive experience in parathyroid surgery, would have better prerequisites for parathyroid localization, identification and management. This is mirrored in the elevated rates of permanent hypoPT in both our study and the study of Reinke et al. (16). As thyroid surgery globally is performed more frequently than parathyroid surgery, there must be a significant number of surgeons who only have experience with thyroid surgery and not parathyroid surgery. Accordingly, the actual rates of permanent hypoPT on a global scale might be significantly underestimated, hereby emphasizing the relevance of our study.

The rate of immediate hypoPT in our study was 37.1% and of these patients, only five (5.6%) recovered, hereby being classified as having transient hypoPT. Thus, we experienced a very high level of irreversible parathyroid damage. This is in contrast to other studies where the majority of patients with immediate hypoPT recovered (1, 2, 31–35). Again, this discrepancy might be a question of definition. Some studies report rates of transient hypocalcemia which is not necessarily the same as transient hypoPT. If we consider postoperative hypocalcemia in our study, the rate of immediate postoperative hypocalcemia would be 64.0%. Many of these patients only had a mild, asymptomatic hypocalcemia that remitted on the usual calcium and vitamin D3 supplementation that was given prophylactically to all patients in accordance with local guidelines. As many of these patients did not receive active vitamin D, they were not classified as having immediate hypoPT, yet they had immediate hypocalcemia. By addressing immediate hypocalcemia instead of immediate hypoPT, more patients would experience recovery and thus be classified as transient.

Other studies (36) consider low PTH levels at POD1 as being indicative of immediate postoperative hypoPT. According to that definition, the rate of immediate hypoPT in our study would be 49.4%, yielding a transient hypoPT rate of 27.9%. Again, this is significantly higher than the 5.6% we report according to the definition chosen in our study. In other words, more patients with immediate PG damage seem to recovery based on that definition. The reason for this discrepancy, is the fact that a significant number of patients with low PTH levels at POD1, were not given active vitamin D, as their values were slightly below 1.6 pmol/L. If P-PTH normalized within a few day, and P-Ca^2+^ also was in range, they were discharged without active vitamin D supplementation. In other words, P-PTH at POD1 is a more sensitive measure of immediate hypoPT. In contrast, the definition based on active vitamin D treatment reflects a more severe immediate hypoPT. Another potential explanation for the low rate of recovery following PG damage could be the low PG identification rate (61.2%) and the high proportions of patients experiencing PG autotransplantation (19.1%) and inadvertent PG excision (21.3%), as all these events are associated with an increased risk of hypoPT (1, 2, 18, 37).

Parathyroid gland autotransplantation seemed to be associated with increased risk of permanent hypoPT, although without statistical significance. However, of the eight patients that experienced PG autotransplantation and had low levels of P-PTH at POD1 as a measure of some degree of immediate hypoPT, only one patient regained sufficient parathyroid function after one year. This finding questions the ability of PG autotransplantation to prevent permanent hypoPT. Nevertheless, it cannot be ruled out that autotransplanted PGs might regain some function on the long term.

Inadvertent PG excision was associated with a significantly increased risk of permanent hypoPT in our study. Likewise, it seemed like Graves’ disease and heavy specimen weight also were associated with an increased risk of permanent hypoPT, although not statistically significant. Other studies have found the same risk factors to be associated with an increased risk of hypoPT (1, 2, 13, 16, 18, 37), and the lack of statistical significance in our study may be due to the small population size.

Interestingly, we found that more patients with permanent hypoPT experienced immediate RLN injury, although not statistically significant. A possible explanation for this finding could be a shift in the surgeon’s attention towards the nerve (and away from the PGs) once a nerve injury has been detected intraoperatively. Furthermore, such injuries normally occur in particularly difficult cases such as Graves’ disease with severe bleeding or a complicated neck due to e.g., high BMI. It can thus be an indicator of a challenging procedure, which by itself could complicate adequate PGs preservation. More studies of a larger magnitude are needed to further enlighten this association.

Altogether, the high rates of hypoPT, PG autotransplantation and inadvertent PG excision, combined with the low PG identification rates found in our study suggest that low-volume, non-parathyroid surgeons might be particularly challenged in finding PG intraoperatively. As a consequence, some studies have suggested the centralization of total thyroidectomy cases to high-volume institutions (23) also experienced in parathyroid surgery (16). However, modification of the surgical technique by using intraoperative autofluorescence-based devices that are able to facilitate intraoperative PG visualization could potentially also be a solution (38). A way of using intraoperative autofluorescence implies the use of a probe containing a camera and a near-infrared light source. During surgery, the surgeon can turn on the probe and point it towards the surgical field. Parathyroid glands will then appear as fluorescing subjects on a screen linked to the fluorescence device, thereby becoming visible to the surgeon. Several studies have shown promising results with higher PG identification rates, early PG identification, reduction in PG autotransplantation and inadvertent PG excision when autofluorescence was used during surgery (22, 34, 39). A future examination of the impact of autofluorescence on hypoPT in low-volume institutions that only perform thyroid surgery would therefore indeed be of interest.

The main limitations of our study consist of the small population size and the retrospective nature of the design. However, as data from low-volume, non-parathyroid institutions are extremely sparse in the literature, we believe that our findings are of significant importance. Furthermore, the patients were identified through different procedure codes for total thyroidectomy. In theory, some patients could have been wrongly coded and therefore missed. However, this seems highly unlikely as the economic compensation for the Department is based on these procedure codes. Accordingly, correct coding is thoroughly controlled making the patient selection process relatively accurate.

In conclusion, both immediate and permanent hypoPT following total thyroidectomy at a low-volume, non-parathyroid institution seems to be much more common than previously reported, reaching very high rates. The elevated rates are most likely due to the low-volume, non-parathyroid nature of the surgeons which in part is mirrored in low PG identification rates, high rates of PG autotransplantation and inadvertent PG excision. Further research evaluating the impact of novel surgical techniques aiming to enhance PG preservation in such institutions is crucial in order to reduce this frequent complication.

## Data availability statement

The raw data supporting the conclusions of this article will be made available by the authors, without undue reservation.

## Ethics statement

The studies involving humans were approved by Institutional Review Board, Goedstrup Hospital. The studies were conducted in accordance with the local legislation and institutional requirements. Written informed consent for participation was not required from the participants or the participants’ legal guardians/next of kin in accordance with the national legislation and institutional requirements.

## Author contributions

AA: Formal analysis, Funding acquisition, Methodology, Project administration, Software, Writing – original draft. TO: Formal analysis, Methodology, Supervision, Writing – review & editing. LR: Formal analysis, Methodology, Supervision, Writing – review & editing. FT: Formal analysis, Methodology, Supervision, Writing – review & editing. PV: Formal analysis, Methodology, Supervision, Writing – review & editing.
